# Predicting the Compressive Strength of Concrete Containing Binary Supplementary Cementitious Material Using Machine Learning Approach

**DOI:** 10.3390/ma15155336

**Published:** 2022-08-03

**Authors:** Nozar Moradi, Mohammad Hadi Tavana, Mohammad Reza Habibi, Moslem Amiri, Mohammad Javad Moradi, Visar Farhangi

**Affiliations:** 1 Department of Civil Engineering, Kermanshah Branch, Islamic Azad University, Kermanshah 6718997551, Iran; nozar.moradi@yahoo.com (N.M.); m.r.habibi@iauksh.ac.ir (M.R.H.); m.amiri@iauksh.ac.ir (M.A.); 2 Department of Civil and Environmental Engineering, Carleton University, Ottawa, ON K1S 5B6, Canada; mjmoradi@cmail.carleton.ca; 3 Department of Civil Engineering, Construction Management and Environmental Engineering, Northern Arizona University, Flagstaff, AZ 86011, USA; visar.farhangi@nau.edu

**Keywords:** machine learning, metakaolin, mechanical property, fly ash, zeolite

## Abstract

Several advantages of supplementary cementitious materials (SCMs) have led to widespread use in the concrete industry. Many various SCMs with different characteristics are used to produce sustainable concrete. Each of these materials has its specific properties and therefore plays a different role in enhancing the mechanical properties of concrete. Multiple and often conflicting demands of concrete properties can be addressed by using combinations of two or more SCMs. Thus, understanding the effect of each SCM, as well as their combination in concrete, may pave the way for further utilization. This study aims to develop a robust and time-saving method based on Machine Learning (ML) to predict the compressive strength of concrete containing binary SCMs at various ages. To do so, a database containing a mixture of design, physical, and chemical properties of pozzolan and age of specimens have been collected from literature. A total of 21 mix design containing binary mixes of fly ash, metakaolin, and zeolite were prepared and experimentally tests to fill the possible gap in the literature and to increase the efficiency and accuracy of the ML-based model. The accuracy of the proposed model was shown to be accurate and ML-based model is able to predict the compressive strength of concrete containing any arbitrary SCMs at ay ages precisely. By using the model, the optimum replacement level of any combination of SCMs, as well as the behavior of binary cementitious systems containing two different SCMs, can be determined.

## 1. Introduction

The increasing demand in consumption of concrete as the second most consumed material in the world, environmental pollution, the need for optimal utilization of materials, and the positive effects of using supplementary cementitious materials (SCMs) on the properties of concrete have led to the widespread use of these materials in the concrete industry. These materials need to have sufficient amorphous aluminosilicates which react with calcium hydroxide in the presence of water to form one or more hydration products: calcium silicate hydrate (C-S-H), calcium aluminate hydrate (C-A-H), and calcium aluminosilicate hydrate (C-A-S-H) [1,2]. Since SCMs have a very small amount of embodied CO${}_{2}$ which is defined based on the total amount of CO${}_{2}$ produced in the extraction and transportation of raw materials and their manufacture into the final product, they are susceptible to producing sustainable concrete [3].

There are several SCMs that each have specific properties and therefore play a different role in enhancing the mechanical properties of concrete, for example, Silica fume shows a significant pozzolanic reaction rate which results in an increase in the compressive strength at early ages [4], and Fly ash has very slow hydration characteristics, thus providing very little contribution to early-age strength [5] but instead providing suitable workability [6]. On the other hand, the ability of SCMs to react with calcium hydroxide is under the influence of the chemical and physical properties of that specific SCM [7]. Therefore, it is essential to know each SCM’s properties to combine the benefits of each supplementary material and minimize their adverse effects. Moreover, multiple and often conflicting demands of concrete properties can be addressed by using combinations of two or more SCMs. A remarkable number of experimental studies have been conducted to evaluate the effect of replacing ordinary Portland cement (OPC) with SCMs such as fly ash (FA), ground granulated blast furnace slag (SL), Metakaolin (MK), rice husk ash (RA), and silica fume (SF), on the mechanical and durability properties of the fresh and hardened concrete. It was shown that when SCMs were used at optimal levels, they can significantly enhance the fresh and hardened state properties of the concrete [8]. The main concern is the optimal level of replacement, especially in binary and ternary cementitious blends.

Several studies have been conducted to determine the effect of adding two or more pozzolan in a mix design. The positive effect of combined usage of SF and FA in pore size distribution [9], compressive strength [9,10,11,12], chloride permeability [6,11,13,14,15,16], alkali-silica reactions [6,17,18], and sulfate resistance [6] have been reported. The experimental study by Shannag [19] indicated that combinations of a certain natural pozzolan with silica fume (SF) can enhance the workability as well as the mechanical properties of concretes, more than natural pozzolan or SF alone. However, high cost and limited availability of SF and construction problems, such as dispersion difficulties and increased water demand, are drawbacks of using this material in dosages much higher than 5% [20]. It was shown in the study of Thomas et al. [6] that the combination of SL and SF can result in a high compressive strength at an early age, even more than the 28-day compressive strength of the control mixture. The same results were obtained by Bleszynski et al. [13]. Moreover, the results of sulfate resistance by Thomas et al. [6] demonstrated that the expansion owing to sulfate attack of the mixture containing type V cement is higher than of mixture including a combination of slag and SF. Almost the same results were determined by Lane and Ozyildirim [17,18] on the expansion of ternary concrete caused by the alkali-silica reaction. Chloride permeability results of Ahmed et al. [15] indicate that the combination of SL and SF has better performance in comparison with the mixtures including SL. This results are in accordance with Thomas et al. [6], Bleszynski et al. [13], and Lee et al. [14] experimental outcomes. The positive effect of a combination of FA and SL in producing concrete with higher compressive strengths compared with the mixtures containing single SCMs was determined in Tan and Pu’s study [21]. The same results were obtained by Li and Zhao [22]. Jianyong and Pei [23] concluded that implementation of both SF and SL results in an improvement in the mechanical properties of concrete. In order to achieve a cost-effective concrete, binary combinations of SL and FA were evaluated by Jeong et al. [24]. It was shown that concrete containing binary SCMs shows satisfactory mechanical properties compared with ordinary mixtures. The results of Radlinski and Olek’s study [25] show that the binary mix design containing SF and FA indicates an enhancement in the compressive strength, chloride permeability, and water absorption compared to results according to the individual effects of FA and SF in a mixture. Most of these studies’ results indicate that the ternary combination of pozzolans has shown superior performance compared to binary mixtures, in terms of the rheological, mechanical, and durability properties of concrete. However, the effect of zeolite (ZE) as one of the suitable SCM to replace cement in binary or ternary mix designs has rarely been evaluated.

Implementation of ZE as an SCM has received significant attention in the past decade owing to the beneficial effects of this SCM on the mechanical properties and durability [26]. This silicate mineral contains large amounts of active AL${}_{2}$O${}_{3}$ and SiO${}_{2}$ in its chemical composition, which causes the conversion of the Ca(OH)${}_{2}$ of the hydration process into C-S-H gel. Moreover, ZE is able to accelerate the process of hydration of cement [27]. However, the variation of the mechanical properties of concrete containing ZE [28,29] and reduction in workability of the mixture [30,31,32,33] are the main drawbacks of widespread utilization of this SCM. Prior research related to the use of ZE in ternary systems is scarce and the knowledge of their performance is limited.

The current study aims to provide a model based on an artificial neural network (ANN) to predict the compressive strength of concrete containing two types of pozzolans. For this purpose, 192 data from previous research have been selected carefully and parameters such as mix design, physical and chemical properties of pozzolan, and age of specimens have been considered as influential factors in the compressive strength of ordinary concrete. In addition, in order to increase the efficiency and accuracy of the proposed model, extensive experimental research was conducted to fill the gaps in the literature. A total of 21 OPC mix designs, including binary mixes, were prepared by substantially replacing cement with FA, MK, and ZE. The compressive strength tests were conducted on concrete mixtures. Moreover, the other goal is to optimize the replacement level of SCMs in ordinary concrete with the objective of achieving comparable mechanical properties. Moreover, being aware of the behavior of binary cementitious systems containing two different SCMs is the other main goal of the present study.

## 2. Artificial Neural Network

Toward the estimation of the compressive strength of ordinary concrete containing two different types of SCMs, a robust approach called Multi-Layer Perceptron (MLP) is used. One of the main benefits of using MLP is the simplification of the utilization and improvement of the accuracy of results [12,34]. Incomplex operating elements that work alongside make MLP function. In nature, the performance of the human brain, which is a neural network, is regulated by the way in which the components are interconnected [35,36]. Thus, it is feasible to develop a simulated structure like natural networks, and obtain the relation among its components by adjusting the weights of each connection. Subsequently, by adjusting the weights of each connection or in other words, training the neural network, applying a particular input results in a specific output. Minimizing the difference between the output and the real result, i.e., target, is the main objective of training. This is done by changing the weight during the learning process and continuing until the error function is less than the specified limit. Training is a repetitious strategy by initializing the weight values, predicting the output of the network, and calculating the corresponding error. Error is relatively high at the first step, since weights are randomly privileged. The main objective of learning in an ML-based method is the acquisition of the weights that leads to the lowest error range. In most artificial networks, the number of weights is high and so there is no direct method to find the weights [37]. Determining weights by trying and error also wastes time and effort. One of the efficient methods to asset the least sets of errors more quickly during network training is the gradient descent approach. Gradient descent, as the name implies, utilizes the error gradient to reduce the error [38,39]. The error is completely affected by the output of the network, and it depends on the weighted output of the hidden neurons, and it depends on the weights. Therefore, by moving toward the input layer and adjusting the weights, the difference between the output of the network and target results may be reduced. This method is known as backpropagation which is a gradient descent algorithm in which the weights of a network move in the opposite direction to the performance function slope. The hidden neurons can compute their error to adjust the weights according to the error signal [34,40].

The following assumptions can be considered for an MLP network:

1. Simple elements known as neurons are responsible for the processing of information,
2. Processed information is passed neuron over connection link,
3. An associated weight is considered for each connection link,
4. Inputs are transmitted from a predefined activation function in neurons and outputs are determined.

The configuration of an MLP network, along with the learning algorithm and the activation function applied in each neuron, is defined as a network. Implementation of the neural networks may decrease the number of experiments and save time and cost [41,42].

### Dataset

**Figure 1:**
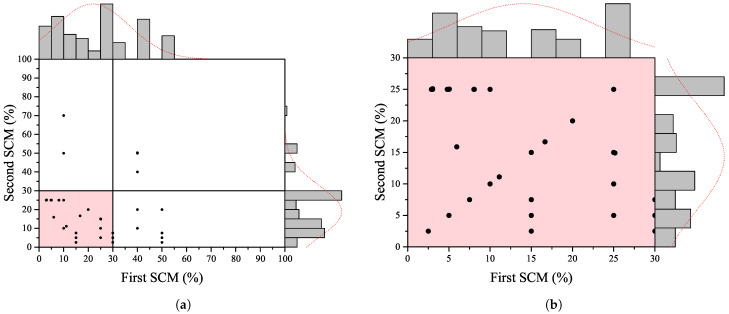
The marginal histogram of binary SCMs percentages (**a**) in the literature, (**b**) in the current dataset.

A deep and careful survey of the literature was done in order to develop a network to estimate the compressive strength of the concrete containing two types of SCMs. The dataset includes about 192 samples with 19 distinguished features. The collected dataset contained information about water content (W), cement (C), fine aggregate (F), coarse aggregate (G), binary SCMs (SCM1 and SCM2), the chemical composition of each SCMs including SiO${}_{2}$, CaO, Fe${}_{2}$O${}_{3}$, Al${}_{2}$O${}_{3}$, MgO, and physical properties of binary SCMs, i.e., specific surface (SS), and age of specimens. The mold of specimens was considered, so the compressive strength was converted to a 150 mm × 300 mm cylinder standard mold. The compressive strength (f${}_{c}$) was considered as the output of the network. Figure 1a shows the marginal histogram of binary SCMs percentages. It is worth noting that any type of pozzolan is considered a SCM as long as its physical and chemical characteristics are known. As can be seen, the numbers of data points indicating the implementation of SCMs of more than 30% are lower. In other words, the main focus of previous studies was on replacing cement with SCMs by 30% of weight or less. Therefore, the dataset is limited to experiments with the maximum usage of 30% for each SCM. This results in reducing the dataset to 142. It is worth noting that Figure 1 indicates the replacement level in percent, while in the proposed network, the effect of SCMs is considered as a replacement weight to fully cover any arbitrary concrete mix design.

**Figure 2:**
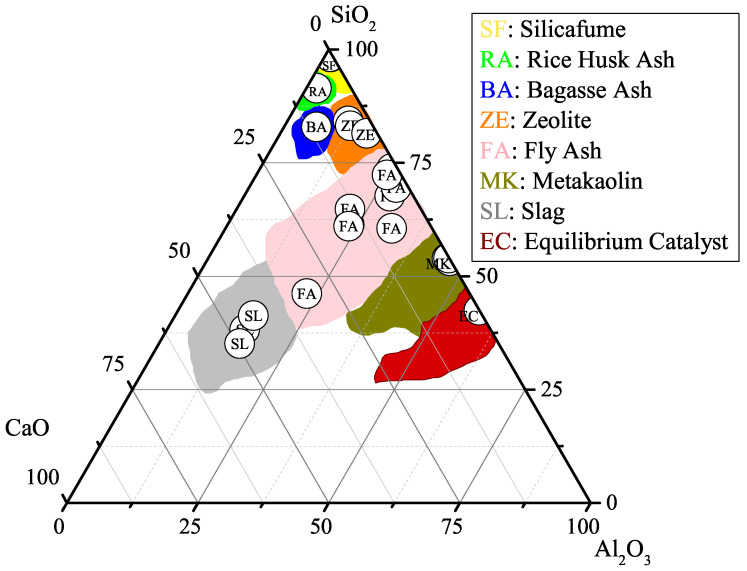
Chemical composition of various SCMs in the dataset.

Furthermore, in order to increase the number of data points in the dataset and fill the possible gap in the previous experimental research, numerous studies were performed in the laboratory. The experimental study was designed to determine the effect of SCMs such as MK, ZE, and FA on different percentages of cement replacement. By doing so, the number of data points increased to 226. Thus, the marginal histogram of binary SCMs in the dataset changed into Figure 1b. Table 1 shows statistical parameters for the dataset. A diverse range of SCMs was investigated in the literature along with the current experimental study. The chemical composition of various SCMs are plotted in Figure 2 on a Al${}_{2}$O${}_{3}$-SiO${}_{2}$-CaO ternary figure. As can be seen, a wide range of pozzolanic materials are considered as the influential parameters on the compressive strength of concrete containing binary SCMs.

## 3. Experimental Program

### 3.1. Material

**Figure 3:**
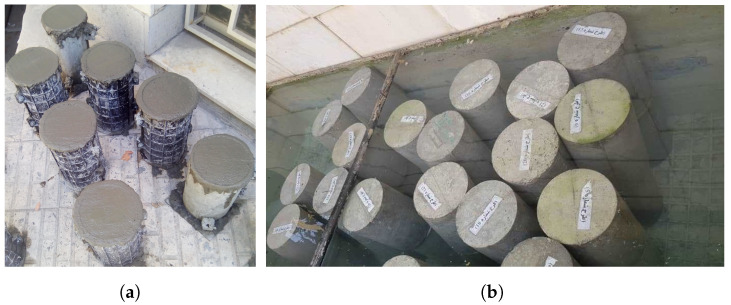
(**a**) Molding the concrete specimens and, (**b**) Curing the cylindrical specimens.

A type I Portland cement with the chemical composition summarized in Table 2 was used in all mixtures. Clean, well-graded, and natural fine and coarse aggregate with unit weights of 2.61 and 2.68, and water absorption of 1.8 and 1.4%, respectively, were used. Tap water was used for making and curing all concrete samples (Figure 3b). Reaching a constant slump in each mix design demands the utilization of a polycarboxylic acid-based high-range water reducer (Carboxal HF5000). The mixing procedure was performed according to the ASTM C192 [43]. A commercially-available MK with chemical properties as shown in Table 3 was procured for use in this study. The FA produced at DRIK company in accordance with the specification listed in Table 4 was used. Natural ZE was provided by a local manufacturer with the chemical specification shown in Table 5.

### 3.2. Mix Proportions and Test Method

In order to understand the effect of binary pozzolan and enrich the collected dataset, 21 distinct mix designs were considered to have a constant water/binder ratio of 0.45 and total binder content of 350 kg/m${}^{3}$. SCMs contain MK+ZE, MK+FA, and FA+ZE, in which a proportion of Portland cement was replaced with the SCMs. The replacement levels for SCMs were up to 50% with 5% intervals. These mix designs are shown in Table 6. The mixture codes were assigned based on the inclusion of pozzolan replacement, i.e., Metakaolin (MK), Zeolite (ZE), and Fly ash (FA). For instance, the mix coded MK5ZE5 was made with 5% metakaolin and zeolite replacement. For each mix design, the compressive strength of concrete was conducted at 3, 7, 28, and 90 days of age. A cylindrical 150 × 300 mm mold was used for the compressive strength test according to ASTM C39 [44] (Figure 3a).

## 4. MLP Modeling

Generally, the procedure of indicating a complex real-world event as a combination of mathematical expressions is called modeling [45]. Governing the suitable network configuration in which the lowest error and highest accuracy can be obtained is vital. To do so, a trial and error procedure is utilized to ascertain the optimal number of neurons in the middle layer, which is called a hidden layer. For each network with a specific number of neurons in the hidden layer, the mean squared error (MSE) indicating the performance of the network is calculated 30 times.

**Figure 4:**
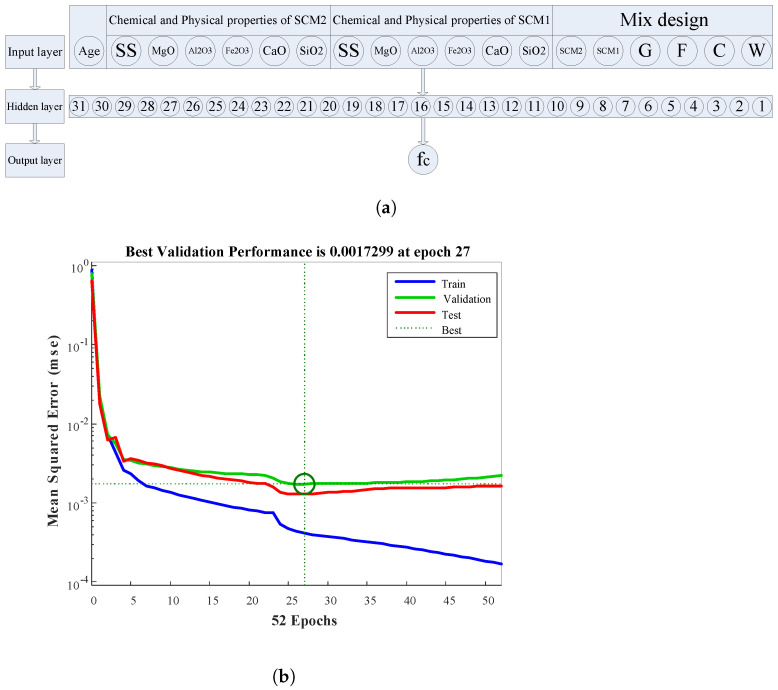
(**a**) The optimal configuration of the MLP network, (**b**) The performance of the proposed MLP network.

The network with the lowest MSE is considered to be an optimum network with a specific number of neurons in the hidden layer. Changing the weights matrix during the training step using an iterative procedure and continuing this until performance reaches the specified goal is the most vital part of network learning. The MSE error in the initial step is relatively high since weights are selected randomly. Finding weights by trial and error that result in the lowest MSE would require a great deal of time and effort [45]. One of the efficient approaches to encountering the least sets of errors within the model learning step is the gradient descent method. Since the error is related to the output of the network, and it depends on the weights, updating weights in each step results in precise outcomes. After some steps, the accuracy of the network for validation data remains constant and the optimal configuration of the network along with its optimum weights matrix will be determined. The optimal configuration of the MLP network, along with its performance, is shown in Figure 4. The most important and effective parameters, such as concrete mix design, physical and chemical properties of both pozzolans, and age, are considered in the proposed network. So long as the MLP network is trained, the compressive strength of concrete containing binary SCMs can be estimated. Training the MLP network was done using the linearly normalized input and by the implementation of the Levenberg–Marquardt (LM) algorithm owing to suitable convergence, high accuracy, and less time consumption [37]. Data is randomly segregated into 3 distinguished parts, namely training, validation, and test. In the proposed MLP network, 70% of data are assigned for training, and two 15% remaining data are considered for validation and test. It was shown that the aforementioned ratio has the best performance [46]. Two commonly used activation functions of TANSIG ($y=\frac{1-e^{-2x}}{1+e^{-2x}}$) and PURELIN (y=x) were used in the hidden and output layer, respectively. Once the desired network performance is obtained, the learning procedure is considered completed.

**Figure 5:**
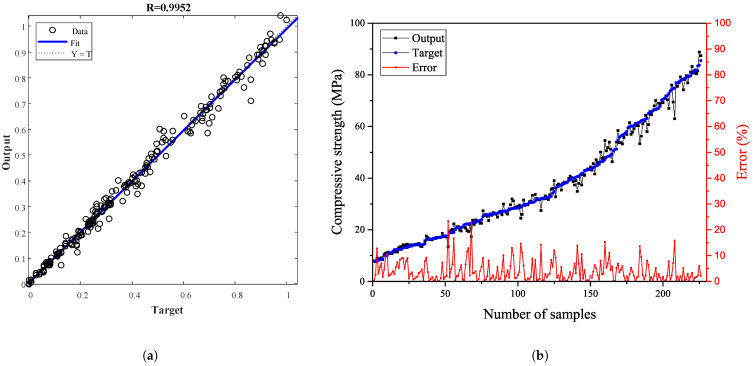
(**a**) The regression of the MLP network, (**b**) The comparison of the predicted outcomes using MLP network and experimental data along with the error.

**Figure 6:**
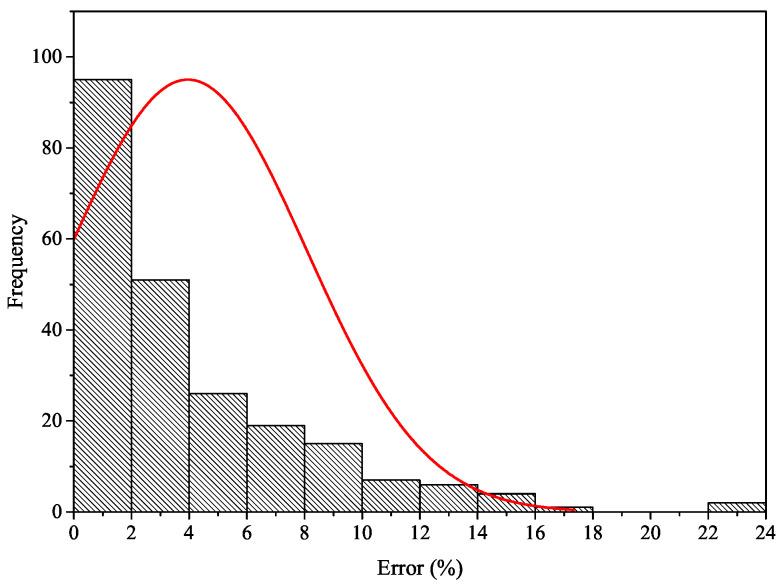
The histogram of error along with distribution curve.

The performance of the MLP networks with reference to predicting the compressive strength of concrete containing binary SCMs is shown in Figure 4b. The best validation performance was acquired as 0.0017 at the 27th epoch. The quality of the prediction as a function of the correlation coefficient, R, for all data is demonstrated in Figure 5a, revealing the correlation between the target (experimental f${}_{c}$) and the MLP network result. The overall response with a correlation coefficient close to 1 verified that the network computed the outcomes with reasonable precision. The comparison of the predicted compressive strength using MLP network (output) and experimental f${}_{c}$ (target) along with MSE of target and output is depicted in Figure 5b. It can be concluded that the network is able to estimate the compressive strength of concrete containing binary SCMs with an acceptable error. The histogram of error is plotted in Figure 6. As it is obvious, more than 42% and 94% of data is predicted with an error of less than 2% and 10%, respectively.

The statistical error values for the estimated compressive strength of concrete containing binary SCMs obtained from the MLP network are described as root mean square error (RMSE), Nash–Sutcliffe efficiency (NSE) coefficient, mean absolute percentage error (MAPE), and correlation coefficient (R). The aforementioned statistical measures can be calculated using Equation (1). These statistical indicators, including MSE, are compared in Table 7 according to all data points. Zero or near to zero are ideal values for all statistical parameters, except for NSE and R, while the ideal value for NSE and R is one. RMSE stipulates the deviation between the experimental results and estimated outcomes of the MLP network. Both the estimation error and the ratio of the error to the experimental value are reflected in MAPE [39,47]. Assessment of the estimation capability of the MLP network is determined using the NSE coefficient. The statistical metrics in Table 7 show that the results of the MLP network in estimating the compressive strengths of concrete containing binary SCMs are close to the experimental results in a satisfactory manner. This further validates the acceptability of the proposed MLP model.

(1) $$\begin{array}{c} RMSE=\sqrt{\frac{\sum {\left(\hat{f_{c}}-f_{c}\right)}^{2}}{N}}\;NSE=1-\frac{\sum {\left(\hat{f_{c}}-f_{c}\right)}^{2}}{\sum {\left(\bar{f_{c}}-f_{c}\right)}^{2}}\\ MAPE=\frac{100}{N}\sum \left|\frac{\hat{f_{c}}-f_{c}}{f_{c}}\right|\;R=\frac{\sum \left(\hat{f_{c}}-\bar{\hat{f_{c}}}\right)\left(f_{c}-\bar{f_{c}}\right)}{\sqrt{\sum {\left(\hat{f_{c}}-\bar{\hat{f_{c}}}\right)}^{2}}\sqrt{\sum {\left(f_{c}-\bar{f_{c}}\right)}^{2}}} \end{array}$$

where $f_{c}$ and $\hat{f_{c}}$ are the experimental compressive strengths and estimated outcomes of the MLP network, and the $\bar{f_{c}}$ and $\bar{\hat{f_{c}}}$ parameters are the averages of the experimental and the estimated values, respectively.

## 5. Results and Discussion

### 5.1. Experimental Compressive Strength

**Figure 7:**
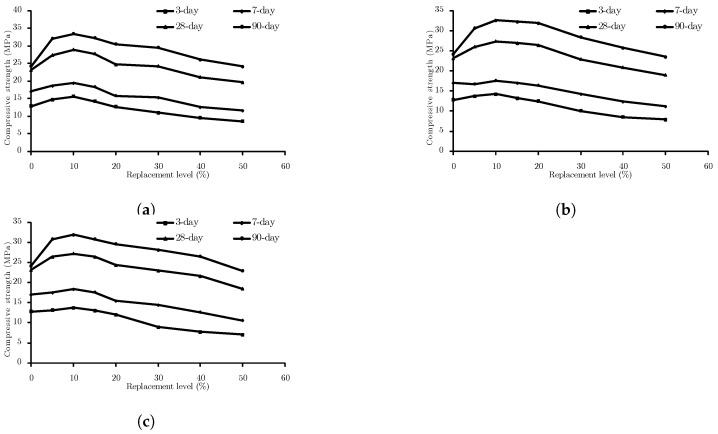
Effect of (**a**) MK-FA, (**b**) MK-ZE, (**c**) FA-ZE on the compressive strength of concrete at different ages.

The experimental results of the compressive strength of specimens with different percentages of pozzolan at various ages are listed in Table 8. The effect of a binary combination of SCMs on the compressive strength of concrete at the age of 3, 7, 28, and 90 days is depicted in Figure 7. MK is shown to be more beneficial in combination with FA considering 15, 22, and 11% increased compared with the control specimen in MK2.5FA2.5, MK5FA5, and MK7.5FA7.5, respectively. This is in accordance with the results of Grist et al. [48]. As can be seen in Figure 7, all the binary mixtures, regardless of the contained pozzolan, have the same optimum replacement level. The optimal replacement level for binary mix design is determined to be 10% of cement weight. Since the effect of SCMs becomes more pronounced with a rise in time, the improvement in 90-day compressive strength is reported herein. The 90-day compressive strength of concrete specimens containing MK and FA has increased by 38% compared to the control specimen at the same age. The amount of enhancement for concrete containing MK and ZE is 35%, and for concrete with ZE and FA ash is 32%.

### 5.2. Prediction of the Compressive Strength

With a focus on estimating the compressive strength of concrete containing binary SCMs, including physical and chemical properties, an MLP network was trained and its performance was assessed. As results indicated, the network could estimate the compressive strength of concrete containing binary SCMs with suitable accuracy which is sufficient in practical use. One of the main advantages of machine learning approaches is the ability to solve complex problems with numerous affecting parameters, especially in the engineering field. As it is discussed, in the current study, there are 19 affecting parameters on the compressive strength of concrete containing binary SCMs. Finding a suitable, accurate, and time-consuming method to estimate the compressive strength according to the inputs is simple thanks to the machine learning approaches. Furthermore, these approaches, after ensuring their accuracy and performance, can be used to produce new results based on new input parameters. This is called generalization, in which a new dataset (unseen data) that is costly or impossible to experiment is fed into the network and results are estimated using a previously-learned machine learning approach.

Since the main objective of the current study is to predict the compressive strength of concrete containing binary SCMs with various chemical and physical properties, the generalization feature of the MLP network will be used. In order to determine the effect of SCMs replacement level, types, and properties, the percentage of substituting cement with SCMs and their pozzolanic characteristics are considered as variables, and the variation in the compressive strength due to changes in these parameters is determined. Utilizing the MLP network outcomes, a wide range of concrete mixtures can be evaluated. Therefore, the concrete mix design is assumed to be constant and the parameters of the mix design are chosen to be around their median [37]. The assumed mix design is summarized in Table 9.

**Figure 8:**
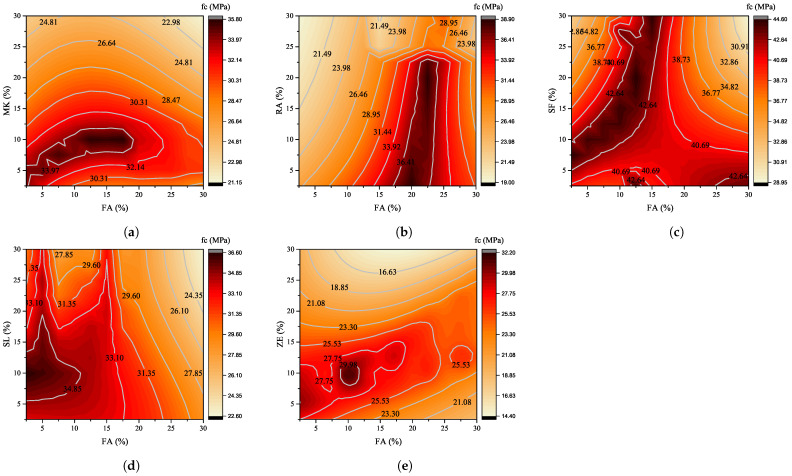
Effect of different percentages of various binary SCMs, (**a**) FA-MK, (**b**) FA-RA, (**c**) FA-SF, (**d**) FA-SL, and (**e**) FA-ZE, on the compressive strength of concrete.

It is worth noting that the proposed MLP network is able to estimate the compressive strength of concrete containing any known or unknown pozzolanic material. In other words, since the proposed network determines the strength of concrete by using the physical and chemical properties of pozzolans, it can also estimate the compressive strength of concrete containing an SCM that may be introduced in the future. In the first step, in order to determine the effect of replacement level, 6 common and well-known SCMs are used to develop new results. The physical and chemical properties of these SCMs are listed in Table 10. The replacement level of SCMs was presumed to be between 2.5 and 30% with 2.5% intervals. In addition, the age of specimens is considered to be at 56 days. The results of MLP network prediction are depicted in Figure 8.

As can be seen, the general trend of variation in the compressive strength of concrete containing binary SCMs indicates that there is an optimum level of replacement for each specific combination of pozzolans. For instance, in a concrete mixture with FA and MK replacement, the maximum compressive strength may be obtained for an FA replacement level of less than 20% and an MK replacement level of less than 12%. Moreover, the effect of an increase in the FA replacement is more significant than the MK percentage. Figure 8b shows the changes in the compressive strength of concrete made with a combination of FA and RA. As can be seen, the effect of RA replacement level in improving the compressive strength is less than FA. The main reason for this trend may be attributed to the higher pozzolanic reactivity of FA compared with RA. The optimum level for this combination of SCMs is around 20% for FA and 2.5 to 20% for RA. This is in accordance with the results of [49,50,51] that consider the improvement as a result of the synergic effect of using binary pozzolans.

According to Figure 8c, it can be said that both SCMs, i.e., FA and SF, are almost equally effective in improving the compressive strength of concrete. The higher reactivity of SF, due to higher surface area and higher amount of SiO${}_{2}$, leads the compressive strength to be further improved in the combination of FA-SF concrete. Higher percentage levels of SF in the presence of the lower amount of FA replacement increases the compression strength of concrete, which is in accordance with the experimental results obtained by [15,52]. For concrete specimens containing FA and SL (Figure 8d), the highest compressive strength is obtained for the replacement level of pozzolans lower than 30%. Moreover, it can be concluded that the pozzolanic effect of both FA and SL is almost the same since the compressive strength in the replacement levels of less than 15% has lower fluctuation. Almost the same results were observed in the experiments of Jeong et al. [24], in which the combined effect of FA and SL at various replacement levels were investigated. It was shown that increasing replacement levels of SL lead to neglectable changes in the compressive strength.

The combined effect of using ZE with other SCMs is rarely studied. In Figure 8e, the effect of implementation of a binary combination of FA-ZE was evaluated using the results of the MLP network. As can be seen, there is an optimum level of replacement in order to reach the maximum compressive strength. The optimal replacement level for both SCMs is around 10% of cement weight. The experimental results (Figure 7c) indicate the suitable replacement level as 5% for each SCMs. The reason for incompatibility may be attributed to the different physical and chemical properties of FA considered in experimental and machine learning approach. However, the fact that there is an optimum replacement level is observed in both experimental and machine learning methods.

The other general trend in Figure 8 is a reduction in the compressive strength in higher replacement levels. In almost all the cases, the minimum compressive strength occurs in 30% replacement level for two studied SCMs. Adding higher amounts of SCMs may result in the dilution effect, which is a reduction in the hydration reaction owing to the lack of sufficient cement content in the mixture [45,53].

**Figure 9:**
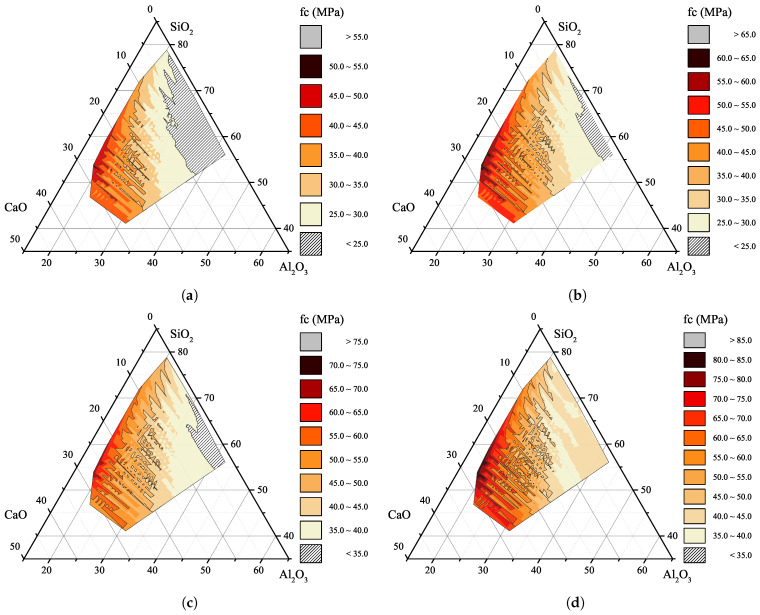
Effect of various combination of chemical composition of SCM on the compressive strength of concrete at (**a**) 28-days, (**b**) 56-days, (**c**) 90-days, and (**d**) 365-days.

In order to understand the effect of the chemical composition of SCMs on the compressive strength of concrete, several predictions are conducted using the proposed MLP network. For better comparison, in these predictions, the concrete mix design, replacement level, and physical characteristics of SCMs are assumed to be constant (Table 11). The mix design is the same as Table 10, the replacement level for both SCMs is considered to be 10%, and the second pozzolan used in the mixture is assumed to be SF and constant during the generalization. Moreover, since the summation of chemical composition of a SCMs need to be 100%, those combinations of SiO${}_{2}$, CaO, and Al${}_{2}$O${}_{3}$ which exceed 93.12%, i.e., (100% − (Fe${}_{2}$O${}_{3}$ + MgO)), is omitted from the generalization outcomes. This may result in 5770 distinguished mix designs at various ages. Changes in the compressive strength of concrete containing binary SCMs against the chemical properties of pozzolans are shown in Figure 9. The empty area in this figure indicates the impossible outcome of the unseen data, i.e., the summation of SiO${}_{2}$, CaO, and Al${}_{2}$O${}_{3}$ exceeds 93.12%. As can be seen, an increase in the age of concrete specimens results in an enhancement in the compressive strength of concrete. This obvious trend once again validates the performance of the MLP network and demonstrates its accuracy in predicting unseen data.

As can be seen from Figure 9a–d, an increase in the amount of SiO${}_{2}$ and CaO and at the same time reduction in the percentage of Al${}_{2}$O${}_{3}$ results in an enhancement in the compressive strength of concrete containing SCMs. This is in accordance with the experimental results of Kasaniya et al. [2]. In their tests, it was shown that level of reactivity of FA depends on the amount of SiO${}_{2}$+CaO+Al${}_{2}$O${}_{3}$ as well as the particle size of SCMs. Furthermore, the regions in Figure 9 with higher compressive strength indicate Class C fly ashes. The comparison between Class F and Class C fly ashes was done by several researchers [54,55,56]. The results of their research confirm the reliability of the outcomes of the MLP network in predicting the compressive strength of concrete containing binary SCMs. Moreover, time plays a vital role in highly reactive pozzolans. In other words, an increase in the compressive strength of concrete containing a highly reactive pozzolan occurs at a higher rate compared with pozzolans with a lower amount of SiO${}_{2}$. The same trend can be observed from Figure 9a–d.

## 6. Developing a Software to Predict the Results

**Figure 10:**
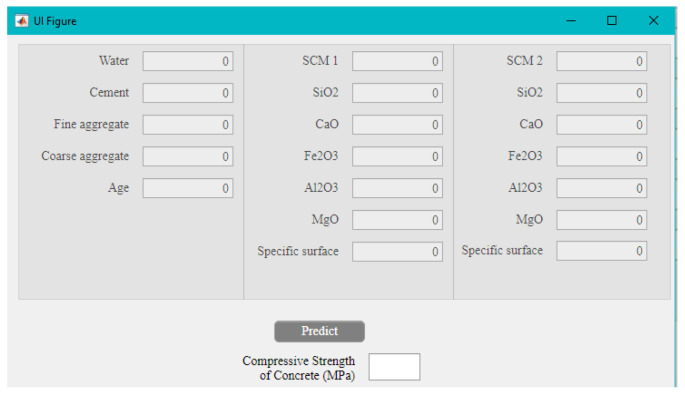
The developed software for predicting the compressive strength of concrete containing binary SCMs.

One of the most suitable and simplest ways to use the results of a machine learning method in practice is to implement the weights obtained from the network in a numerical system and in the form of user-friendly software. This may be achieved by using a graphical user interface (GUI) in the Matlab environment. With such an approach and using the developed software, there is no need to perform complex and time-consuming calculations, and by implementing the optimal weights obtained from the network, the results can be estimated with appropriate accuracy. This may help engineers to achieve the results without conducting experimental tests or computing numerous complex equations. Figure 10 demonstrates the main GUI. As can be seen, the compressive strength of concrete containing binary SCMs at any age between 3 to 365 can be estimated by considering the concrete mix design, along with the chemical composition of pozzolans.

## 7. Conclusions

The current experimental study was carried out to fill the existing gap in the literature on the evaluation of the compressive strength of concrete containing binary SCMs. The effect of various replacement levels of three different common pozzolans, namely Metakaolin, Zeolite, and Fly ash in concrete mixtures, was done. It was shown that the optimal replacement level for binary mix design is determined to be 10% of cement weight.

In addition, an accurate and comprehensive database of previous research on the effect of using binary pozzolans on the compressive strength of concrete was collected. The database contains 19 important factors on the compressive strength of concrete containing binary SCMs. Using the MLP method, an attempt was made to develop a comprehensive, reliable, and accurate model for predicting the compressive strength of concrete containing binary SCMs. The accuracy of the model in terms of MSE was 0.0017 for validation data. Furthermore, more than 42% and 94% of data were predicted with an error of less than 2% and 10%, respectively.

By ensuring the accuracy of the proposed model, the unseen results based on the generalization technique were obtained. The effect of various combinations of SCMs with any arbitrary chemical composition in any age between 3 to 365 days can be predicted with high accuracy using the MLP network proposed in this paper. In order to show the capability of the MLP network, several simulations were done and the results were compared with the proven fact and previous experimental tests. The outcomes of the MLP network demonstrate a reliable precision in estimating unseen data and can be used for further prediction of any concrete mixture with any combination of SCMs. Finally, to facilitate the utilization of the proposed MLP network, user-friendly software was developed based on the prediction procedure of the machine learning method. The proficiency and competence of this tool has been successfully proven. 

## Figures and Tables

**Table 1:** Statistical parameters for the concrete containing binary SCMs dataset.

Attribute	Unit	min	max	Average	Standard Deviation
Water	kg/m${}^{3}$	155	216	172	16.8
Cement	kg/m${}^{3}$	175	476	291	76.5
Fine Aggregate	kg/m${}^{3}$	470	971	798	163.5
Coarse Aggregate	kg/m${}^{3}$	865	1268	963.5	80.3
First SCM	kg/m${}^{3}$	8.75	126	54.3	33.6
SiO${}_{2}$	%	36	96	54	11.7
CaO	%	0.09	38.1	6.6	11.5
Fe${}_{2}$O${}_{3}$	%	0.46	13.8	3	4.5
Al${}_{2}$O${}_{3}$	%	0.1	45	28.8	16
MgO	%	0.01	6.6	1.7	2.5
Specific Surface	cm${}^{2}$/kg	3100	235,000	17,316	36,623
Second SCM	kg/m${}^{3}$	8.75	165	62	46.5
SiO${}_{2}$	%	31.5	94.9	62.5	21.8
CaO	%	0.06	44.4	9.7	12.5
Fe${}_{2}$O${}_{3}$	%	0.45	13.4	4.1	3.3
Al${}_{2}$O${}_{3}$	%	1	54.5	14.8	9.2
MgO	%	0.01	6.8	3.1	2.2
Specific Surface	cm${}^{2}$/kg	3100	1,500,700	92,688.8	178,892.4
Age	days	3	365	39.9	55.4
Compressive strength	MPa	7.9	85.5	37.7	21.6

**Table 2:** Physical and chemical specification of the Portland cement.

**Chemical** **Specification**	**SiO** ${}_{2}$	**Al**${}_{2}$O${}_{3}$	**Fe**${}_{2}$O${}_{3}$	**CaO**	**MgO**	**SO** ${}_{3}$	**K** ${}_{2}$ **O**	**Na** ${}_{2}$ **O**	**L.O.I**	**I.R**	**C** ${}_{3}$ **S**	**C** ${}_{2}$ **S**	**C** ${}_{3}$ **A**	**C** ${}_{4}$ **AF**
	21.6	5.8	3.1	61.4	4	1	0.6	0.21	2	0.2	47.24	25.02	8.26	12.1
**Physical** **Specification**	**Specific** **surface [m${}^{2}/$kg]**			**Specific** **gravity [kg/m${}^{3}$]**			**Initial Setting** **Time [min]**			**Final Setting** **Time [min]**			**28-day compressive** **strength [MPa]**	
	350			3120			100			195			49	

**Table 3:** Physical and chemical specification of MK.

**Chemical** **Specification**	**SiO** ${}_{2}$	**Al** ${}_{2}$ **O** ${}_{3}$	**Fe** ${}_{2}$ **O** ${}_{3}$	**CaO**	**MgO**	**TiO** ${}_{2}$	**K** ${}_{2}$ **O**	**Na**${}_{2}$O	**L.O.I**
	52.3	45.1	0.7	0.08	0.03	0.69	0.03	0.02	1.05
**Physical** **Specification**	**Specific** **surface [m${}^{2}/$kg]**		**Specific** **gravity [kg/m${}^{3}$]**		**PH**	**color**		**Humidity [%]**	
	2500		2600		4–5	white		0.5–1	

**Table 4:** Physical and chemical specification of FA.

**Chemical** **Specification**	**SiO** ${}_{2}$	**Al** ${}_{2}$ **O** ${}_{3}$	**Fe** ${}_{2}$ **O** ${}_{3}$	**CaO**	**MgO**	**K** ${}_{2}$ **O**	**Na** ${}_{2}$ **O**	**L.O.I**
	48.5	29.75	7.8	6.62	1.78	0.03	0.38	1.86
**Physical** **Specification**	**Specific** **surface [m${}^{2}/$kg]**		**Specific** **gravity [kg/m${}^{3}$]**		**PH**	**color**	**Humidity [%]**	
	600		2300		4–5	light gray	5–8	

**Table 5:** Physical and chemical specification of ZE.

**Chemical** **Specification**	**SiO** ${}_{2}$	**Al** ${}_{2}$ **O** ${}_{3}$	**Fe** ${}_{2}$ **O** ${}_{3}$	**CaO**	**MgO**	**TiO** ${}_{2}$	**K** ${}_{2}$ **O**	**Na** ${}_{2}$ **O**	**L.O.I**
	67.79	13.66	1.44	1.68	1.2	0.21	3.12	0.02	10.88
**Physical** **Specification**	**Specific** **surface [m${}^{2}/$kg]**		**Specific** **gravity [kg/m${}^{3}$]**		**PH**	**color**		**Humidity [%]**	
	1800		2350		4–5	yellow		5–8	

**Table 6:** Details of mix proportions [All unites are in kg/m${}^{3}$].

Mixture	Cement	Water	Fine Aggregate	Coarse Aggregate	MK	ZE	FA
MK2.5ZE2.5	332.5	160	971	912	8.75	8.75	0
MK5ZE5	315	160	971	912	17.5	17.5	0
MK7.5ZE7.5	297.5	160	971	912	26.3	26.3	0
MK10ZE10	280	160	971	912	35	35	0
MK15ZE15	245	160	971	912	52.5	52.5	0
MK20ZE20	210	160	971	912	70	70	0
MK25ZE25	175	160	971	912	87.5	87.5	0
MK2.5FA2.5	332.5	160	971	912	8.75	0	8.75
MK5FA5	315	160	971	912	17.5	0	17.5
MK7.5FA7.5	297.5	160	971	912	26.3	0	26.3
MK10FA10	280	160	971	912	35	0	35
MK15FA15	245	160	971	912	52.5	0	52.5
MK20FA20	210	160	971	912	70	0	70
MK25FA25	175	160	971	912	87.5	0	87.5
ZE2.5FA2.5	332.5	160	971	912	0	8.75	8.75
ZE5FA5	315	160	971	912	0	17.5	17.5
ZE7.5FA7.5	297.5	160	971	912	0	26.3	26.3
ZE10FA10	280	160	971	912	0	35	35
ZE15FA15	245	160	971	912	0	52.5	52.5
ZE20FA20	210	160	971	912	0	70	70
ZE25FA25	175	160	971	912	0	87.5	87.5

**Table 7:** Comparison of five statistical measures to validate the performance of the MLP network.

Output	MSE	RMSE	NSE	MAPE	R
Compressive strength	$7.43^{-4}$	0.0272	0.9903	7.24%	0.9952

**Table 8:** Compressive strength of specimens [All units are in MPa].

Mixture	3-Day	Compare with Control Specimen	7-Day	Compare with Control Specimen	28-Day	Compare with Control Specimen	90-Day	Compare with Control Specimen
Control	12.8	–	17	–	23.1	–	24.2	–
MK2.5ZE2.5	13.7	1.07	16.7	0.98	26	1.13	30.6	1.26
MK5ZE5	14.2	1.11	17.6	1.04	27.3	1.18	32.6	1.35
MK7.5Z7E.5	13.2	1.03	17	1.00	26.9	1.16	32.2	1.33
MK10ZE10	12.4	0.97	16.4	0.96	26.4	1.14	31.9	1.32
MK15ZE15	10	0.78	14.2	0.84	22.9	0.99	28.4	1.17
MK20ZE20	8.5	0.66	12.3	0.72	20.8	0.90	25.7	1.06
MK25ZE25	7.9	0.62	11.1	0.65	18.9	0.82	23.5	0.97
MK2.5FA2.5	14.7	1.15	18.6	1.09	27.3	1.18	32	1.32
MK5FA5	15.6	1.22	19.4	1.14	28.9	1.25	33.4	1.38
MK7.5FA7.5	14.2	1.11	18.3	1.08	27.7	1.20	32.2	1.33
MK10FA10	12.6	0.98	15.8	0.93	24.7	1.07	30.5	1.26
MK15FA15	11	0.86	15.3	0.90	24.2	1.05	29.5	1.22
MK20FA20	9.5	0.74	12.5	0.74	21.1	0.91	26.1	1.08
MK25FA25	8.5	0.66	11.5	0.68	19.6	0.85	24.1	1.00
ZE2.5FA2.5	13.1	1.02	17.5	1.03	26.4	1.14	30.8	1.27
ZE5FA5	13.7	1.07	18.3	1.08	27.2	1.18	31.9	1.32
ZE7.5FA7.5	13	1.02	17.6	1.04	26.4	1.14	30.8	1.27
ZE10FA10	12	0.94	15.4	0.91	24.3	1.05	29.6	1.22
ZE15FA15	8.9	0.70	14.4	0.85	23	1.00	28.1	1.16
ZE20FA20	7.7	0.60	12.6	0.74	21.6	0.94	26.5	1.10
ZE25FA25	7	0.55	10.5	0.62	18.4	0.80	22.9	0.95

**Table 9:** The assumed mix design of concrete (All units in (kg/m${}^{3}$)).

Mixture	Cement	Water	Coarse Aggregate	Fine Aggregate	First SCM	Second SCM
Assumed Mix Design	350	160	912	971	8.75–105	8.75–105

**Table 10:** The physical and chemical properties of SCMs used to generate new results.

SCM	SiO${}_{2}$ (%)	CaO (%)	Fe${}_{2}$O${}_{3}$ (%)	Al${}_{2}$O${}_{3}$ (%)	MgO (%)	Specific Surface (cm${}^{2}$/g)
FA	57.64	12.01	4.45	19.23	2.43	3100
MK	51.37	0.23	0.46	44.6	0.03	3950
RA	76.3	5.5	1.5	1.6	0.01	11,000
SF	94.9	0.5	0.7	1	0.61	153,000
SL	31.55	44.38	0.53	13.79	5.2	4497
ZE	67.79	1.68	1.44	13.66	1.2	18,000

**Table 11:** The variation of the chemical properties of SCMs used to generate new results.

SCM	Replacement Level (kg/m${}^{3}$)	SiO${}_{2}$ (%)	CaO (%)	Fe${}_{2}$O${}_{3}$ (%)	Al${}_{2}$O${}_{3}$ (%)	MgO (%)	Specific Surface (cm${}^{2}$/g)
Variation of pozzolans	35	42 to 76.5	1 to 31	4.45	17 to 38	2.43	3100
SF	35	94.9	0.5	0.7	1	0.61	153,000

## Data Availability

The data presented in this study are available on request from the corresponding author.

## Abbreviations

The following abbreviations are used in this manuscript. The order is based on their appearance:

SCM	Supplementary Cementitious Material
ML	Machine Learning
C-S-H	Calcium Silicate Hydrate
C-A-H	Calcium Aluminate Hydrate
C-A-S-H	Calcium Alumino Silicate Hydrate
OPC	Ordinary Portland Cement
FA	Fly Ash
SL	Furnace Slag
MK	Metakaolin
RA	Rice Husk Ash
SF	Silica Fume
ZE	Zeolite
BA	Bagasse Ash
EC	Equilibrium Catalyst
ANN	Artificial Neural Network
MLP	Multi-Layer Perceptron
W	Water
C	Cement
F	Fine aggregate
G	Coarse aggregate
SS	Specific Surface
f${}_{c}$	compressive strength
MSE	Mean Squared Error
LM	Levenberg-Marquardt
RMSE	Root Mean Square Error
NSE	Nash-Sutcliffe Efficiency
MAPE	Mean Absolute Percentage Error
R	Correlation Coefficient
